# Pro-oncogenic cytokines and growth factors are differentially expressed in the post-surgical wound fluid from malignant compared to benign breast lesions

**DOI:** 10.1186/s40064-015-1260-8

**Published:** 2015-09-05

**Authors:** Amanda Valeta-Magara, Raheleh Hatami, Deborah Axelrod, Daniel F. Roses, Amber Guth, Silvia C. Formenti, Robert J. Schneider

**Affiliations:** Department of Microbiology, NYU School of Medicine, New York, NY 10016 USA; Department of Surgery, NYU School of Medicine, New York, NY 10016 USA; NYU Perlmutter Cancer Institute, NYU School of Medicine, New York, NY 10016 USA; Department of Radiation Oncology, NYU School of Medicine, New York, NY 10016 USA; Weill Cornell Medical College, New York, NY USA

**Keywords:** Breast cancer, Seroma fluid, Malignancy, Cytokines, Surgical cavity

## Abstract

**Purpose:**

The accumulation of wound fluid known as seroma in the chest cavity following breast surgery is a common occurrence that can persist for many weeks. While the pro-inflammatory composition of seroma is well established, there has been remarkably little research to determine whether seroma contains pro-oncogenic factors, and whether this is influenced by previous malignant disease.

**Methods:**

We developed a clinical trial in which we obtained post-surgical seroma fluids from women with benign or malignant disease 1 or 2 weeks following lumpectomy or mastectomy. We conducted an analysis of more than 80 different cytokines, chemokines and growth factors.

**Results:**

We found that surgical cavity seroma from breast cancer patients has a higher expression of key tumor-promoting cytokines and lower expression of important tumor-inhibiting factors when compared to benign lesions from non-cancer patients. Patients with high body mass index also had higher levels of leptin regardless of malignancy.

**Conclusions:**

We conclude that the breast post-surgical tumor cavity contains factors that are pro-inflammatory regardless of malignant or benign disease, but in malignant disease there is significant enrichment of additional pro-oncogenic chemokines, cytokines and growth factors, and reduction in tumor-inhibiting factors. These results are consistent with tumor conditioning of surrounding normal stromal tissue and creation of a pro-oncogenic environment that persists long after surgical removal of the tumor.

**Electronic supplementary material:**

The online version of this article (doi:10.1186/s40064-015-1260-8) contains supplementary material, which is available to authorized users.

## Background

Post-operative accumulation of seroma in the surgical cavity following breast cancer surgery varies in incidence from 2.5 to 51 % of patients (Siegel et al. 2015). In breast cancer, seroma is defined as serous fluid that collects in the dead space of the post-mastectomy skin flap, axilla, or breast following breast-conserving surgery (Agrawal et al. 2006). Seroma formation is a common consequence of surgical excision, whereby empty space devoid of breast tissue elicits an inflammatory wound healing response with subcutaneous accumulation of serous fluid (Agrawal et al. 2006). Analysis of seroma has shown that it is an inflammatory exudate, classically seen in the first phase of wound repair (McCaul et al. 2000).

While most seroma research is focused on the association with increased infection or its effect on cosmesis (Mukesh et al. 2012), there has been little research on the potential pro-oncogenic composition of seroma fluid itself. Rather, correlations have been made between wound healing and tumor development, supported by observations that chemically initiated or oncogene-expressing cells can develop tumors more efficiently when transplanted into hospitable wound-healing stroma than in non-wound stroma (Hofer et al. 1998). Moreover, one study found that patients with wound complications at primary surgery had an increased risk of systemic recurrence of breast cancer (Murthy et al. 2007). It has also been shown that both overall survival and distant metastasis-free survival are significantly reduced in patients whose tumors express a wound-response signature compared to tumors that do not express this signature (Chang et al. 2005). There are various mechanisms by which wounding can promote tumor development. Among these possibilities, seroma fluid has been shown to possess biological activities such as the ability to increase cancer cell proliferation, migration and invasion (Belletti et al. 2008; Tagliabue et al. 2003). The cytokines, chemokines and growth factors in seroma fluid that regulate these biological activities, however, have not been well characterized.

It is well established that tumors interact with and alter the surrounding stroma, and that such interactions are critical for tumor progression (Junttila and de Sauvage 2013). Given that seroma is derived from the wound-healing response of tumor-adjacent stroma, we asked whether seroma derived from the excision of benign tumors differs from that of malignant tumors, as malignant and benign tumors may activate or influence the adjacent stroma and infiltrating immune cells differently. Here we show that surgical cavity seroma from breast cancer patients has a higher expression of certain tumor-promoting cytokines, including GRO, ENA-78/CXCL5 and TIMP-2, and lower expression of tumor-inhibiting cytokines IGFBP-1, IL-16, IFN-γ, IL-3 and FGF-9, when compared to seroma from non-cancer patients.

## Patients and methods

### Patients and sample collection

Post-surgical seroma fluids from women who had undergone either lumpectomy or mastectomy breast surgery were collected at week 1 or 2 post-surgery by percutaneous aspiration. The clinical protocol (NYU 09-0031) received institutional review board approval at NYU-Langone Medical Center and Bellevue Hospital. All patients provided signed informed consent to participate in the study and seroma fluid for the protocol. The study was opened in February 2010 and closed to accrual in September 2013. A total of 296 patients were accrued to this study, however only 79 developed a clinically detectable and symptomatic seroma by week 1 or 2 post-surgery. Of these patients, in 59 cases an adequate fluid sample was obtained to conduct the proposed molecular studies. After pathological examination, 24 patients were classified as having benign disease (fibroadenomas or benign microcalcifications). The remaining 35 patients had post-surgical pathologically confirmed malignant disease (invasive ductal carcinoma, lobular carcinoma). Patients were classified as ER/PR positive based on a cut off score of ≥2 % by IHC staining. Table 1 describes the clinical and pathologic characteristics of patients who participated in this study. After collection, seroma fluids were centrifuged at 1500 rpm for 10 min at 4 °C to remove debris. Supernatants were aliquoted and stored at −80 °C until analysis.

**Table 1:** Patient characteristics

	Benign		Malignant	
	No.	(%)	No.	(%)
No.	24	40.7	35	59.3
Age, years				
<55	19	79.2	8	22.9
≥55	5	20.8	27	77.1
Tumor size	N/A			
pT1 (≤2 cm)			28	80
pT2 (2–5 cm)			5	14.3
pT3 (>5 cm)			2	5.7
Lymph node status	N/A			
Negative			29	85.3
Positive			5	14.7
Histological grade	N/A			
1			6	18.2
2			18	54.5
3			9	27.3
Estrogen receptor status	N/A			
Negative			2	5.9
Positive			32	94.1
Progesterone receptor status	N/A			
Negative			4	11.8
Positive			30	88.2
HER2 status	N/A			
0			5	15.2
1+			22	66.7
2+			5	15.2
3+			1	3

### Antibody-based protein array system

All 59 seroma specimens were analyzed three times using RayBio™ Human Cytokine Array V membranes (Norcross, GA, USA, cat. # AAH-CYT-5) according to manufacturer instructions. The array simultaneously detects 80 different cytokines, chemokines and growth factors by a quantitative immunoblot sandwich assay. Cytokine array membranes were incubated in blocking buffer for 30 min at room temperature and subsequently incubated with 1 ml of undiluted seroma fluid for 2 h at room temperature. Membranes were washed in washing buffer and incubated with 1 ml of biotin-conjugated detection antibodies overnight at 4 °C. Thereafter, membranes were washed and incubated with 2 ml of streptavidin-horseradish peroxidase at room temperature for 2 h. Membranes were developed using enhanced chemiluminescence, visualized on X-ray film at multiple exposures in the linear sensitivity range (Genemate, catalog no. F-9023-8X10), scanned and spots quantified using densitometric software Image J (NIH).

### Array data normalization and statistical analyses

Positive control signals on each array were used for signal normalization according to manufacturer instructions. Graphing and statistical tests were performed using GraphPad Prism 6.0 software (San Diego, CA, USA). *P* values between two groups (benign, malignant) were calculated using an unpaired Student *t*-test. Multiple group analyses used one-way ANOVA and Tukey multiple comparison tests. Correlation of cytokine, chemokine and growth factor expression with clinical-pathological parameters was determined using Pearson’s correlation analysis. A *P* value of <0.05 was considered to be statistically significant. All data are presented as mean ± SEM. Cellular processes associated with factors present in seroma fluid were determined using the NIH David (database for annotation, visualization and integrated discovery) bioinformatics tool.

## Results

### Study population

Post-surgical seroma was collected from patients undergoing first time breast surgery for benign lesions or malignant tumors. Descriptive characteristics of patients are provided in Table 1. There were 24 patients with benign disease (40.7 % of patients, average age 46 years) and 35 patients with malignant disease (59.3 % of patients, average age 63 years). Malignant tumor size stage distribution was 80 % pT1, 14.3 % pT2, and 5.7 % pT3. Lymph node status was established for all but one patient with malignant disease: 14.7 % lymph node positive, 85.3 % lymph node negative. Histological tumor grade was established for all but two patients with malignant disease: 18.2 % grade 1, 54.5 % grade 2, and 27.3 % grade 3. The majority of malignant tumors were ER/PR positive (94.1 and 88.2 %, respectively), and Her2 negative (81.9 %).

### Factors in common in seroma from benign and malignant lesions

**Fig. 1:**
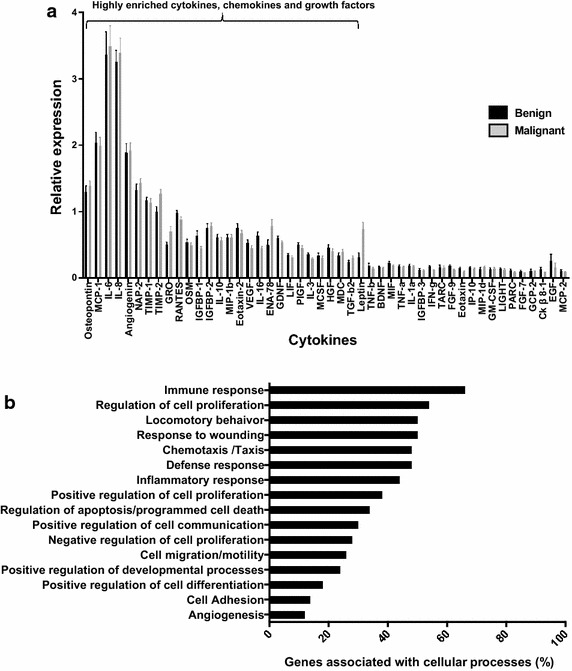
Cytokines, chemokines and growth factors, associated with the wound healing response signature are highly expressed in both benign and malignant breast seroma fluid. **a** Quantification of array membranes. Mean levels from three independent studies of cytokines, chemokines and growth factors expressed in seroma fluid from benign (n = 24) vs. malignant (n = 35) patients are shown. **b** Functional analysis of cytokine, chemokine, and growth factor profiles from benign and malignant post-surgical seroma. Functional analysis indicates seromas are associated with wound healing related cellular processes

The signal intensity for each factor was quantified for all patient seroma samples using Image J densitometry analysis the array data were normalized based on the average positive control signal intensity for each array. The mean signal intensities of every spot were further corrected for local background effects. The collective data of factor expression profiles from benign (n = 24) and malignant (n = 35) seroma specimens are shown (Fig. 1a). From the 80 cytokines that were assayed, 28 were highly expressed in seroma fluid derived from both benign and malignant lesions (Fig. 1a). The most highly expressed cytokines common to both benign and malignant seromas were IL-6, IL-8, MCP-1/CCL2, angiogenin, osteopontin, NAP2/CXCL7, TIMP-1, TIMP-2, RANTES/CCL5, IGFBP-1, IGFBP-2, GRO, OSM, eotaxin-2/CCL24, IL-10, MIP-1β/CCL4, VEGF, ENA-78/CXCL5, GDNF, LIF, PIGF, IL-3, IL-16, MCSF, HGF, MDC/CCL22, TGF-β2 and leptin.

To establish the functional relevance of these enriched factors in seroma fluid, results were subjected to Gene Ontology and Pathway analysis using NIH David Software. The majority of factors in common defined cellular processes associated with wound healing and inflammatory responses in both benign and malignant seroma (Fig. 1b). However, the profile present in post-surgical seroma determined here also contains factors with established tumor-promoting biological effects regardless of original benign or malignant disease. These factors include IL-6, IL-8 and MCP-1/CCL2 that have been shown to possess strong pro-proliferative and pro-oncogenic biological activities (Balkwill and Mantovani 2001).

### Malignant seroma has higher expression of tumor-promoting cytokines and lower levels of tumor-inhibiting factors than benign seroma

**Fig. 2:**
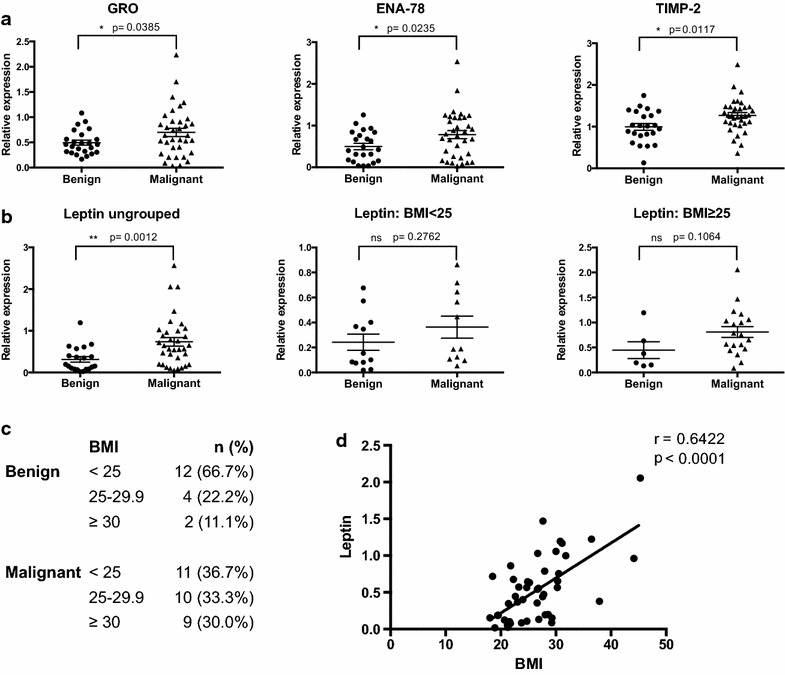
Factors significantly up-regulated in malignant vs. benign seroma profiles. **a** Relative expression of cytokines up-regulated in malignant post-operative seroma fluid compared to benign seroma. **b** Leptin expression in benign and malignant seroma fluid presented collectively and as BMI-matched groups. **c** Distribution of BMI among patients in benign and malignant groups. **d** Pearson correlation analysis showing a significant positive correlation of leptin expression with BMI [Pearson r = 0.6422; p < 0.0001, n = 48 (all benign and malignant seroma samples where data is available)]

**Fig. 3:**
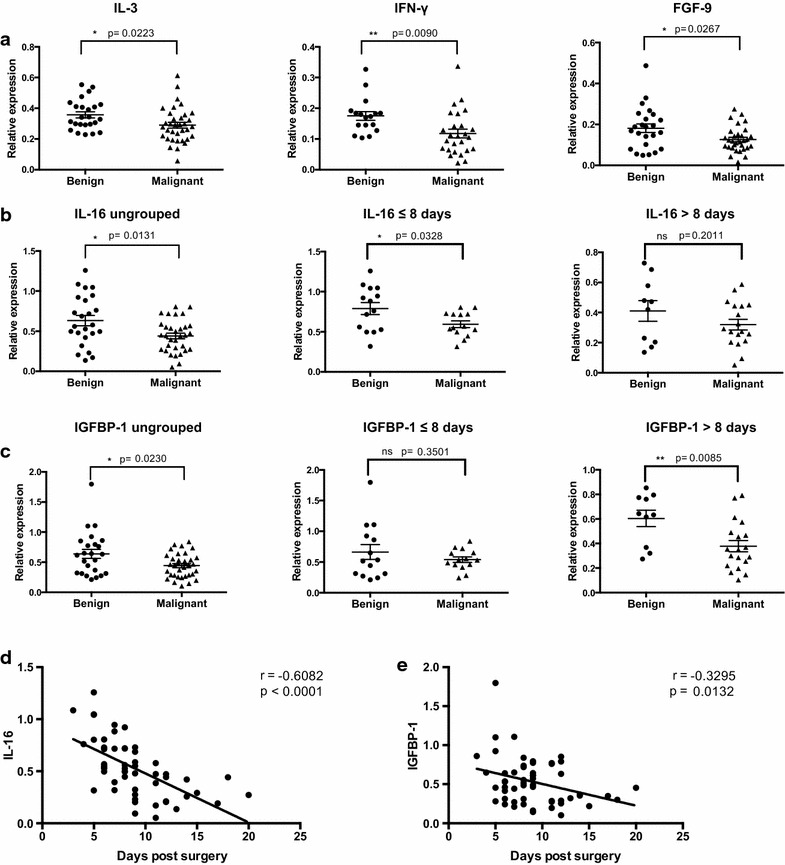
Factors down-regulated in malignant vs. benign seroma profiles. **a** Relative expression of cytokines down-regulated in malignant post-operative seroma fluid compared to benign seroma. **b** IL-16 expression in benign and malignant seroma fluid presented collectively and at specific collection time-points post surgery (group 1 = collection time point ≤8 days and group 2 = collection time-point >8 days). **c** IGFBP-1 expression in benign and malignant seroma fluid presented collectively and at specific collection time-points post surgery (group 1 = collection time point ≤8 days and group 2 = collection time-point >8 days). **d** Pearson correlation analysis showing a significant negative correlation of IL-16 expression and collection time-point of seroma post-surgery [Pearson r = −0.6082; p < 0.0001, n = 54 (all benign and malignant seroma samples where data is available)]. **e** Pearson correlation analysis showing a significant negative correlation of IGFBP-1 expression and collection time-point of seroma post-surgery [Pearson r = −0.3295; p = 0.0132, n = 56 (all benign and malignant seroma samples where data is available)]

Next, we established whether factors were differentially expressed between seroma fluid from malignant versus benign post-surgical cavities. Of the 80 factors assayed, 9 biologically important factors were differentially expressed. In particular, growth-regulated protein (GRO), ENA-78/CXCL5, TIMP-2, and leptin were strongly up-regulated in malignant seroma, whereas IGFBP-1 (insulin-like factor binding protein-1), IL-3, IFN-γ, FGF-9 and IL-16 were down-regulated in malignant versus benign seroma fluid (Figs. 2a, b,  3a–c). Cytokine comparisons between the benign and malignant groups were cross-analyzed with non-tumor associated variables that might affect cytokine expression and were also differentially expressed between the two groups. These were examined as independent variables associated with factor differences. These included association between factor levels and body mass index (BMI), and the time point of seroma collection post-surgery (Table 1; Fig. 2c), tumor grade, HER2 status and patient age. Since 94 % of malignant disease patients were ER+, that could not be studied as an independent variable.

Leptin expression has been well established to correlate positively with high BMI (Monti et al. 2006). Leptin was elevated in malignant seroma samples compared to benign seromas (Fig. 2b). However, BMI distribution was not equal between benign and malignant patients, with a greater number of high BMI patients (BMI ≥25) in the malignant rather than the benign group (63.3 and 33.3 % respectively) (Fig. 2c). We investigated whether the differential expression of leptin between the two groups was dependent or independent of BMI. A Pearson correlation analysis of leptin expression and BMI for all seroma specimens was performed, demonstrating a significant positive correlation between leptin levels and BMI (r = 0.6422, p < 0.0001; Fig. 2d). None of the other factors showed a significant correlation with BMI (Additional file 1: Fig. S1). We therefore analyzed leptin levels between benign and malignant seromas in BMI-matched groups, separating the seroma samples into two groups, normal weight (BMI <25) and overweight plus obese (BMI ≥25). When re-analyzed in this manner, there were no significant differences in leptin expression between the BMI-matched benign and malignant seroma samples, indicating that elevated leptin levels are not independently correlated with malignant diagnosis (Fig. 2b).

We also determined whether time of seroma collection post-surgery influenced cytokine expression. A Pearson correlation analysis compared expression of each of the nine factors identified above and the time of seroma collection post-surgery. Seven of the nine factors did not show a significant correlation between their expression and time of seroma collection and therefore could be analyzed collectively (Additional file 1: Fig. S2). However, IL-16 and IGFBP-1 did demonstrate time-dependent expression, both showing a significant decrease in expression with increased interval of time post-surgery (IL-16: r = −0.6082, p < 0.0001 and IGFBP-1: r = −0.3295, p = 0.0132) (Fig. 3d, e). Consequently, for IL-16 and IGFBP-1, seroma samples were separated into two groups—those collected within the first week post-surgery, likely corresponding to the inflammatory and proliferation phases of wound healing (≤8 days, n = 14 benign, n = 14 malignant) and those collected after the first week post-surgery, likely to correspond to the tissue remodeling phase of wound healing (>8 days, n = 10 benign, n = 18 malignant). With this adjustment it was found that IL-16 was significantly down-regulated in malignant seroma compared to benign seroma collected within the first week post-surgery (p = 0.0328), a difference not observed in seroma collected at later time points (p = 0.2011) (Fig. 3b). IGFBP-1 was significantly down-regulated in seroma from malignant lesions compared to seroma from benign lesions collected after the first week post-surgery (p = 0.0085), a difference not seen in seroma collected within the first week (p = 0.3501) (Fig. 3c).

In summary, having accounted for variables that might introduce bias in the results of the comparison of cytokine expression between post-surgical seroma samples derived from benign and malignant patients, three factors (GRO, ENA-78, TIMP-2) were found to be up-regulated (Fig. 2a) and five factors (IGFBP-1, IL-3, IFN-γ, FGF-9, IL-16) were down-regulated (Fig. 3a–c) in seroma fluid from breast cancer patients compared to non-cancer patients. The well-established biological activities of these differentially expressed cytokines might causally link them to local recurrence and metastasis at the breast tumor surgical wound-site. In fact, the cytokines that were up-regulated in malignant versus benign seroma samples have all been shown to orchestrate key tumor-promoting biological activities in breast cancer (Table 2). Moreover, cytokines down-regulated in malignant compared to benign seromas orchestrate various tumor-inhibiting biological activities (Table 2).

**Table 2:** Characteristics of cytokines/growth factors differentially expressed between benign and malignant seroma fluid

Cytokine/growth factor	Signaling pathways	Physiological function	Function in breast cancer
Up-regulated in malignant vs. benign			
GRO	JAK/STAT, PI3K/AKT, MAPK, PLC/PKC (KEGG Kyoto Encyclopedia of Genes and Genomes 2015)	Attracts and activates granulocytes (Owen and Mohamadzadeh 2013)	Supports growth of triple-negative breast cancer cells (Hartman et al. 2013) Involved in cancer chemo-resistance and metastasis (Acharyya et al. 2012)
ENA-78/CXCL5	JAK/STAT, PI3K/AKT, MAPK, PLC/PKC (KEGG Kyoto Encyclopedia of Genes and Genomes 2015)	Chemoattractant and activator of neutrophil function (Owen and Mohamadzadeh 2013)	Promotes breast cancer cells migration, invasion (Hsu et al. 2013) Regulates cancer stem cells (Liu et al. 2011)
TIMP-2	–	Inhibits metalloproteinase activity (Ellerbroek and Stack 1999) Facilitates cell-surface activation of pro-MMP-2 (Lu et al. 2004; Shen et al. 2010)	TIMP-2 regulates MMP-2-mediated breast cancer cell transmigration through lung microvascular endothelial cells (Shen et al. 2010) High levels of TIMP-2 correlate with adverse prognosis in breast cancer (Remacle et al. 2000)
Down-regulated in malignant vs. benign			
IGFBP-1	–	Binds to IGFs and can either inhibit or stimulate their growth promoting effects (Lee et al. 1993)	Inhibits IGF-mediated breast cancer proliferation (Yee et al. 1994) Inhibits breast cancer cell motility (Zhang and Yee 2002)
IL-3	JAK/STAT, MAPK, PI3K/AKT NF-κB (Reddy et al. 2000)	Haemopoietic growth factor which stimulates the production and functional activity of various blood cell types (Korpelainen et al. 1996)	Increases tumor immunogenicity-promotes macrophage infiltration and promotes development of tumor reactive cytotoxic T-cells^a^ (Pulaski et al. 1996)
IFN-γ	JAK/STAT (Schroder et al. 2004)	Important in host defense mechanisms Has antiviral, immunoregulatory and proinflammatory activities (Schroder et al. 2004)	Inhibits cell growth (Gooch et al. 2000)
FGF-9	MAPK, PI3K/AKT, PLC/PKC (Goetz and Mohammadi 2013)	Important for the regulation of embryonic development, cell proliferation, differentiation and migration (Goetz and Mohammadi 2013)	Induces cancer stem cell expansion (Fillmore et al. 2010)
IL-16	Ca^2+^, PIP_3_, SAPK (Krautwald 1998)	Chemotactic for CD4^+^ T lymphocytes, monocytes, and eosinophils Promotes T-cell proliferation (Wilson et al. 2004)	Promotes cell proliferation^a^ (Atanackovic et al. 2012)

^a^Demonstrated in other cancer types, function in breast cancer not yet established

### Relationship between cytokines and clinical-pathological features

We assessed whether there are statistically significant associations between the eight differentially expressed cytokines and specific clinical-pathological features in the malignant seroma patient group. No statistically significant correlations were found between any of the eight factors and common clinical-pathological features examined, including tumor grade and HER2 status (Additional file 1: Figs. S3, S4), although certain trends were noted. For instance, ENA-78 trended towards increased expression with increasing tumor grade (Additional file 1: Fig. S3) and IL-3 trended towards decreased expression with increasing Her2 overexpression (Additional file 1: Fig. S4).

## Discussion

Seroma fluid has been shown to promote various pro-tumorigenic processes such as cancer cell proliferation, migration, invasion and acquisition of stem-like phenotypes (Belletti et al. 2008; Segatto et al. 2014). This raises the possibility that tumor-promoting biological factors might exist in seroma fluid, although a comprehensive analysis of the cytokines, chemokines and growth factors present in seroma fluid has not yet been conducted. Using single marker ELISA assays, several studies showed that breast seroma fluid contains inflammatory cytokines IL-6, IL-2 and IL-1β (Al-Gaithy 2013). To conduct a more exhaustive analysis, a profile of the proteins present in seroma fluid derived from benign and malignant breast lesions was obtained using cytokine array that characterizes 80 different cytokines, chemokines and growth factors simultaneously. We have shown that irrespective of benign or malignant tumor type, breast seroma fluid is highly enriched in factors that are involved in wound healing, inflammatory responses and tissue remodeling processes. Since seroma fluid is a product of the wound healing response this result was expected. However, our studies have identified a panel of factors present in breast seroma fluid, which have not been previously established. These findings give a plausible explanation for the tumor-promoting biological effects of seroma fluid, as it is highly enriched with many factors such as IL-6, IL-8 and MCP-1/CCL2 that have been shown to possess strong pro-proliferative and pro-oncogenic biological activities (Balkwill and Mantovani 2001).

Of the eight cytokines that were differentially expressed between seroma fluid from breast cancer and non-cancer patients, three were specifically up-regulated in malignant seroma fluid (GRO, ENA-78/CXCL5 and TIMP-2). These factors have been demonstrated to promote various pro-tumorigenic activities in breast cancer, implicating a potential importance of these factors in local recurrence and metastasis of malignant breast tumors. GRO chemokines which consist of three isoforms GRO-α, GRO-β and GRO-γ, also known as CXCL1, CXCL2 and CXCL3 are established granulocyte chemo-attractants (Owen and Mohamadzadeh 2013). The GRO factors have recently been shown to orchestrate tumor-promoting biological activities in breast cancer. For instance, GRO chemokines support the growth of triple-negative breast cancer cell lines (Hartman et al. 2013) and promote breast cancer metastasis and resistance to chemotherapy (Acharyya et al. 2012). ENA-78/CXCL5 is closely related to the GRO chemokines, as they are all ELR^+^CXC chemokines characterized by a Glu-Leu-Arg (ELR) motif preceding the CXC sequence and signal via a common receptor CXCR2 (Strieter et al. 1995). Similar to GRO chemokines, ENA-78/CXCL5 chemo-attracts and activates neutrophils (Owen and Mohamadzadeh 2013) and has also been shown to possess tumor-promoting biological activities. For example, in breast cancer, ENA-78/CXCL5 promotes breast cancer cell migration and invasion (Hsu et al. 2013), and is reported to stimulate cancer stem cell (CSC) activity (Liu et al. 2011). ENA-78 has been associated with poor prognosis, tumor progression, cell proliferation, migration and invasion in other cancer types as well (Kawamura et al. 2012; Li et al. 2011).

Tissue inhibitor of metalloproteinases-2 (TIMP-2) was also specifically up-regulated in malignant breast seroma samples in our study. TIMP-2 is known to regulate matrix metalloproteinase-2 (MMP-2), which plays a role in extra-cellular matrix proteolysis (Ellerbroek and Stack 1999). Traditionally thought of as an MMP-2 inhibitor, it has been demonstrated that TIMP-2 actually plays a dual role in regulating MMP-2 activity and can either initiate or inhibit its activity (Lu et al. 2004; Shen et al. 2010). Due to its dual roles, TIMP-2 has been reported to have both pro-tumorigenic and anti-tumorigenic roles in cancer. For example, by inhibiting the activity of MMP-2, TIMP-2 can block tumor cell invasion (Albini et al. 1991) thereby acting an anti-tumorigenic factor. However, several studies have also highlighted the pro-tumorigenic activities of TIMP-2. TIMP-2 was shown to promote breast tumor metastasis by regulating MMP-2-mediated breast cancer cell transmigration through lung microvascular endothelial cells (Shen et al. 2010). Moreover, using a human glioblastoma cell line model, it was demonstrated that up-regulation of TIMP-2 promotes MMP-2 activation and subsequent glioblastoma cell invasion (Lu et al. 2004). In melanoma cells TIMP-2 over-expression is sufficient to increase NF-κB activity and protect cells from apoptosis (Sun and Stetler-Stevenson 2009). Furthermore, high levels of TIMP-2 are correlated with adverse prognosis in breast cancer (Remacle et al. 2000). In our study, TIMP-2 was more elevated in malignant seroma samples than benign samples, but its significance is still to be determined. Mechanistic studies are needed to define the role of TIMP-2 in breast cancer seroma.

We found five cytokines that were down-regulated in seroma fluid from malignant versus benign lesions (IGFBP-1, IL-3, IFN-γ, FGF-9 and IL-16). Most of these cytokines have been shown to possess anti-tumorigenic properties. Their down-regulation in malignant seroma could conceivably be relevant to the risk of local recurrence and metastasis in malignancies. IGFBP-1 is one of six homologous proteins that specifically bind to insulin-like growth factor (IGF)-I and IGF-II (we also analyzed IGFBP-2, 3 and 4 and their expression was not different between benign and malignant seroma samples). The binding of IGFBP-1 to IGFs prevents their interaction with their receptors, and inhibits the mitogenic and metabolic actions of IGFs (Lee et al. 1993). Consistent with its physiological function, in breast cancer IGFBP-1 has also been shown to inhibit IGF-mediated cell proliferation (Yee et al. 1994) and breast cancer cell motility (Zhang and Yee 2002). Our data supports the findings of Subramanian et al. (2007), who showed that IGFBP-1 mRNA levels were significantly lower in malignant breast tissue compared to normal breast tissue samples (protein levels were not examined). Given that IGFBP-1 possesses tumor suppressive roles in breast cancer, its down-regulation in malignant seroma suggest that it might be involved in promoting malignant tumor progression.

Interleukin-3 (IL-3), also known as multi-lineage-colony stimulating factor (Multi-CSF), is a cytokine that supports the growth and differentiation of a broad range of hematopoietic cells types including hematopoietic stem cells, macrophages and lymphoid cells and enhances immune responses during disease and infection (Korpelainen et al. 1996). In accordance with its established physiological roles, IL-3 was found to increase tumor immunogenicity in mouse models of lung cancer and fibrosarcoma by promoting development of tumor reactive cytotoxic T cells and by promoting infiltration and activation of anti-tumorigenic macrophages (Pulaski et al. 1996). A role for IL-3 in breast cancer has not yet been established. However, our study found that IL-3 was down-regulated in malignant seroma samples compared to benign seromas. Given the importance of IL-3 in augmenting immune responses, it is possible that its down-regulation in malignant seromas might reflect a mechanism by which invasive breast cancers escape immune surveillance.

IFN-γ is a pleiotropic cytokine that is involved in nearly all immune and inflammatory responses including growth, differentiation and activation of T-cells, B-cells, macrophages and natural killer (NK) cells (Schroder et al. 2004). IFN-γ has anti-tumorigenic activities, including tumor immune-surveillance (Dunn et al. 2006; Kaplan et al. 1998), tumor cytostatic/cytotoxic effects (Chawla-Sarkar et al. 2003) and decreases tumor angiogenesis (Beatty and Paterson 2001). With respect to breast cancer, IFN-γ has anti-proliferative effects on breast cancer cell lines (Gooch et al. 2000). It has also been shown that IFN-γ-induced signaling is reduced in B-cells isolated from breast cancer patients compared to those from healthy controls, and impaired IFN-γ signaling was evident in early and late state stage breast cancers (Critchley-Thorne et al. 2009). The well-established role of IFN-γ as an anti-tumorigenic cytokine is consistent with our data demonstrating its down-regulation in malignant versus benign seromas.

FGF9 is a growth factor that is involved in a variety of biological processes including embryonic development, cell proliferation, differentiation and migration (Goetz and Mohammadi 2013). The role of FGF-9 in breast cancer has not been well established, although one study found that estrogen could expand the breast CSC population through a paracrine mechanism that involved FGF9 (Fillmore et al. 2010). We found that FGF9 is down-regulated in malignant compared to benign seroma, a puzzling finding. However, the function of FGF9 has not been well characterized in most cancers, and FGF9 could well have dual roles, as both a pro-and anti-tumorigenic factor whose function could be context/tissue dependent. Further studies are needed to establish the role of FGF-9 in breast seroma.

Interleukin-16 (IL-16) is a CD4^+^ T-cell chemo-attractant that also promotes T-cell proliferation, modulates inflammatory and immune responses and promotes B-cell differentiation (Wilson et al. 2004). The role of IL-16 in cancer has been well studied in hematological malignancies, where its levels are elevated compared to healthy individuals (Atanackovic et al. 2012; Richmond et al. 2011). IL-16 is growth-promoting in multiple myeloma and cutaneous T-cell lymphoma (Atanackovic et al. 2012; Richmond et al. 2011). However, IL-16 function in solid tumors has not been studied. Given that IL-16 is involved in T-cell recruitment and proliferation and modulates other immune responses, it might promote breast tumor immune surveillance and function as a tumor suppressor, accounting for its down-regulation in breast cancer seroma. Further studies are needed to establish the role of IL-16 in breast seroma.

## Conclusion

In conclusion, we conducted an exhaustive analysis of the cytokines, chemokines and growth factors present in benign and malignant breast seroma fluid, showing that after surgery the cavity is enriched in factors involved in wound healing, inflammation and tissue remodeling processes. Moreover, some of the cytokines, chemokines and growth factors enriched in seroma fluid are known enhancers of tumor development and progression. Future studies should be conducted with a larger cohort of patients undergoing breast surgery for benign and malignant lesions to confirm these findings. The differential expression of the eight factors between benign and malignant seroma fluid offers research hypotheses to be explored further to determine their role in breast tumor progression, local recurrence and metastasis.

## Additional file

Additional file 1:
**Figure S1.** Correlation between cytokine expression and BMI in post-surgical seroma fluid. Pearson correlation analysis showing there was no relationship between expression of any these cytokines and BMI. **Figure S2.** Correlation between cytokine expression and collection time-point of seroma post-surgery. Pearson correlation analysis showing there was no relationship between expression of any these cytokines and seroma time of collection post-surgery. **Figure S3.** Comparison of cytokine expression and tumor grade in seroma fluid derived from patients in the malignant group. There were no significant associations between cytokine expression and tumor grade for all the factors studied. **Figure S4.** Comparison of cytokine expression and HER2 status in seroma fluid derived from patients in the malignant group. There were no significant associations between cytokine expression and HER2 status for all the factors studied.
