# Supplementary material for: Pro-oncogenic cytokines and growth factors are differentially expressed in the post-surgical wound fluid from malignant compared to benign breast lesions
Source: Springerplus. 2015 Sep 5;4:483. doi: 10.1186/s40064-015-1260-8 (PMC4560730; doi:10.1186/s40064-015-1260-8)
Supplement: Additional file 1: — Figure S1. Correlation between cytokine expression and BMI in post-surgical seroma fluid. Pearson correlation analysis showing there was no relationship between expression of any these cytokines and BMI. Figure S2. Correlation between cytokine expression and collection time-point of seroma post-surgery. Pearson correlation analysis showing there was no relationship between expression of any these cytokines and seroma time of collection post-surgery. Figure S3. Comparison of cytokine expression and tumor grade in seroma fluid derived from patients in the malignant group. There were no significant associations between cytokine expression and tumor grade for all the factors studied. Figure S4. Comparison of cytokine expression and HER2 status in seroma fluid derived from patients in the malignant group. There were no significant associations between cytokine expression and HER2 status for all the factors studied. [file 40064_2015_1260_MOESM1_ESM.pdf]

Valeta-Magara et al., Suppl. Figure 1

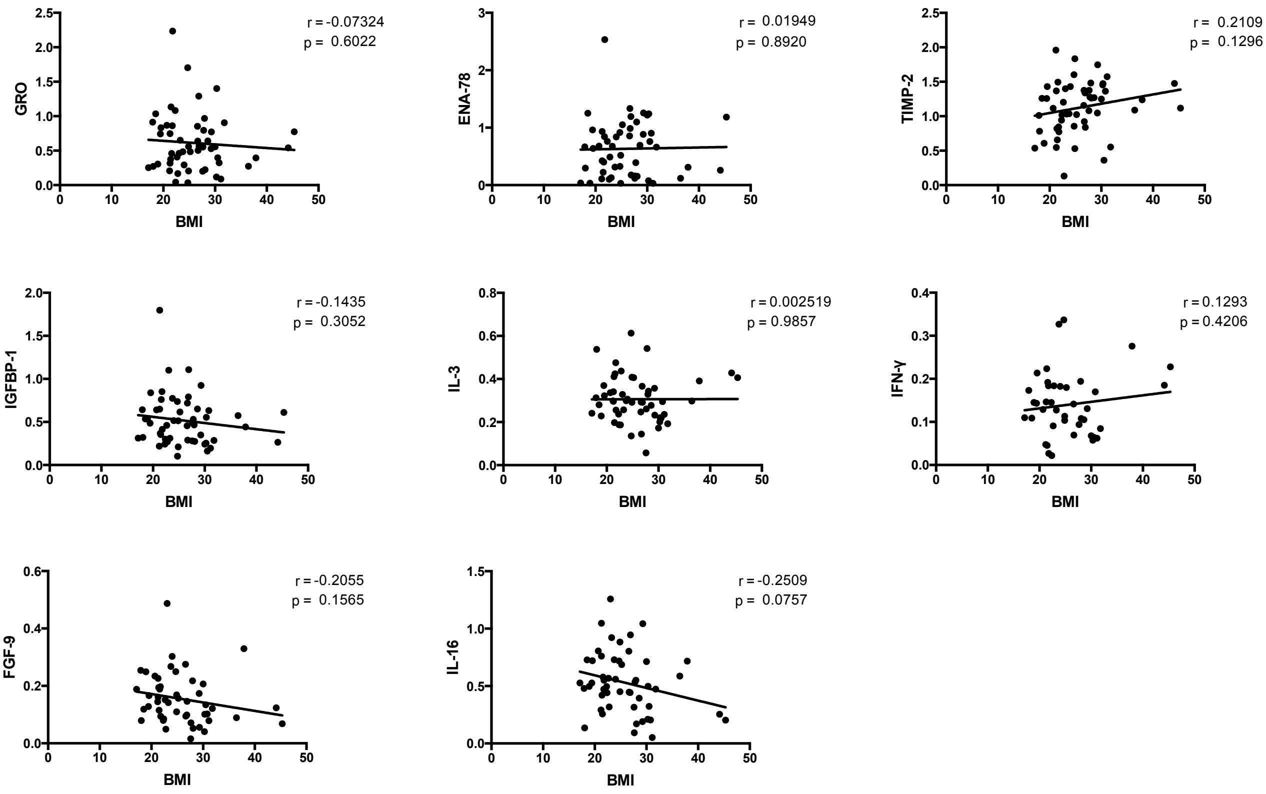

Valeta-Magara et al., Suppl. Figure 2

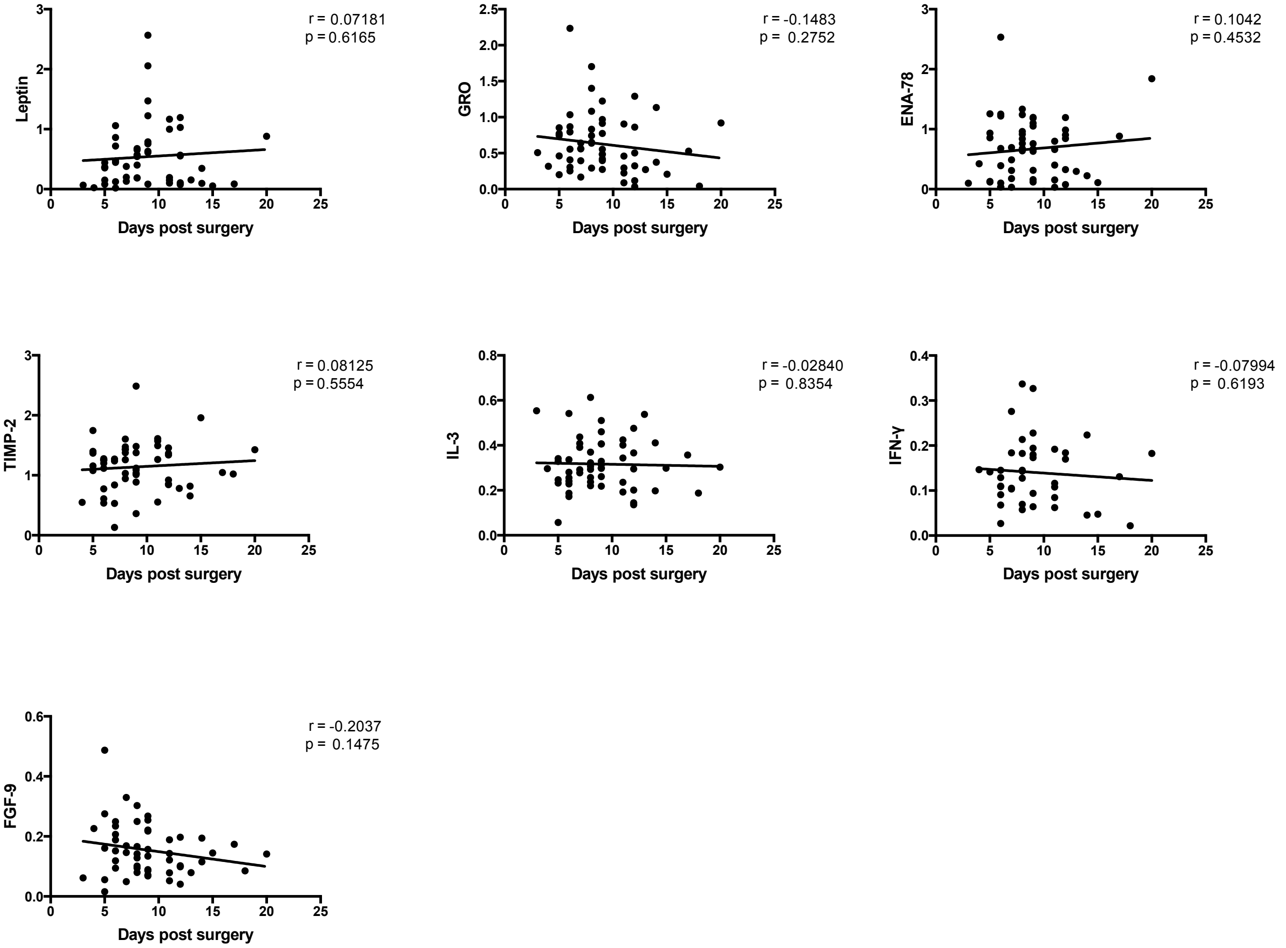

Valeta-Magara et al., Suppl. Figure 3

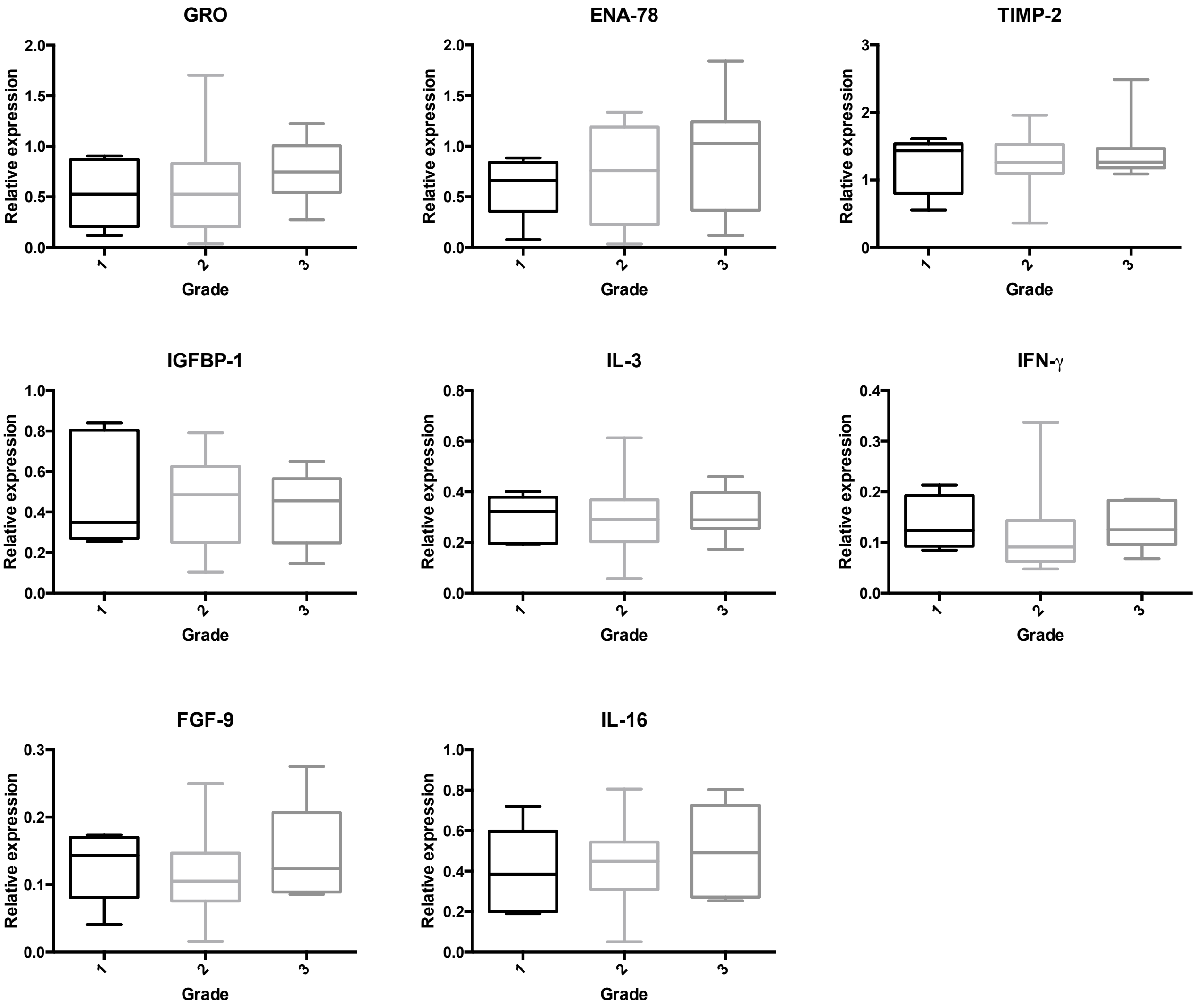

p-value summary from one-way Anova, Tukey's multiple comparisons test

Cytokine	1 vs. 2	1 vs. 3	2 vs. 3
Leptin	0.5994	0.2113	0.4925
GRO	0.8889	0.6338	0.7822
ENA-78	0.9079	0.4800	0.5467
TIMP-2	0.9686	0.6447	0.6378
IGFBP-1	0.8984	0.7763	0.9231
IL-3	0.9994	0.9792	0.9520
IFN-γ	0.9419	0.9955	0.9623
FGF-9	0.9415	0.8617	0.5646
IL-16	0.9112	0.6505	0.7866

Valeta-Magara et al., Suppl. Figure 4

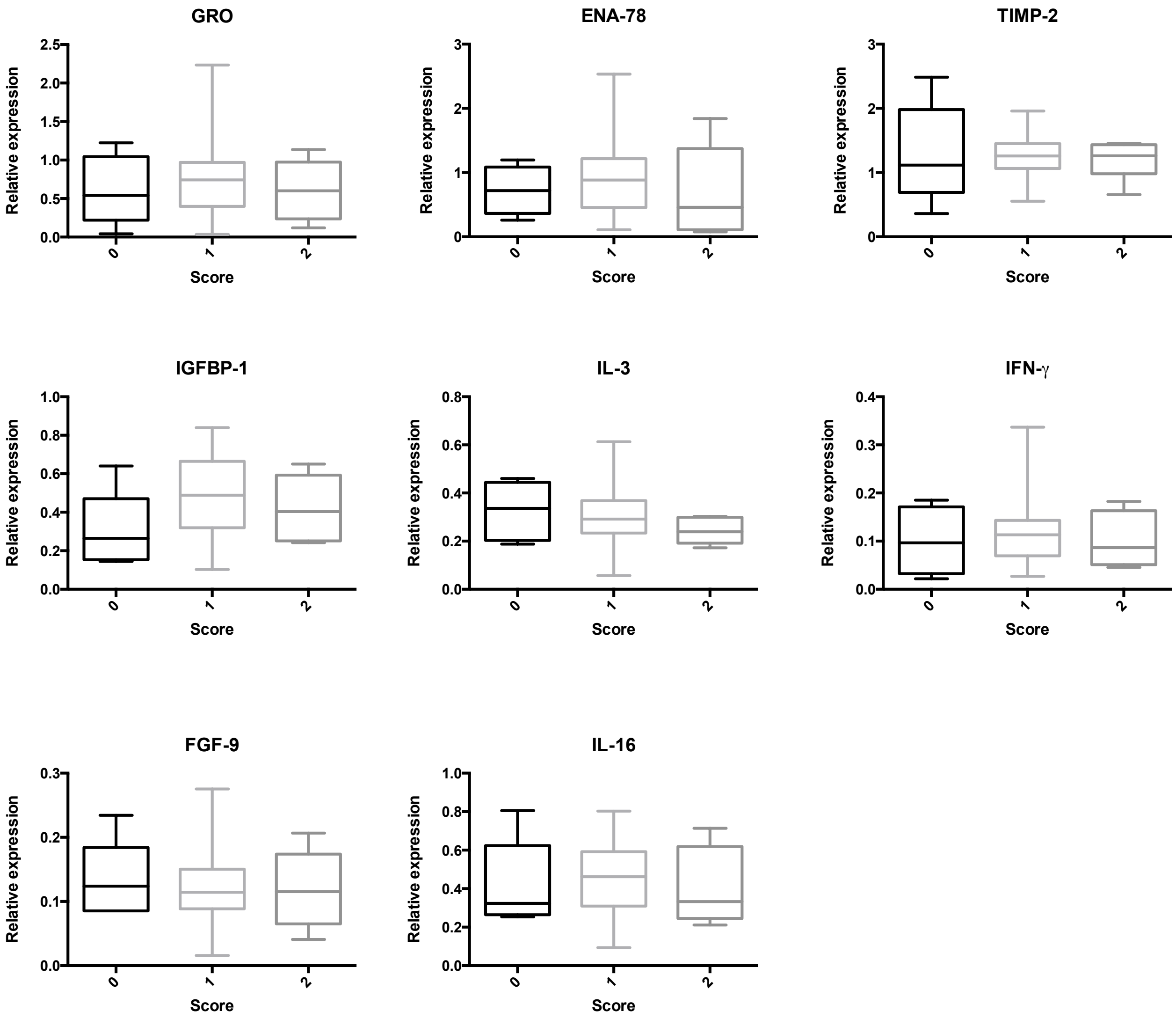

p-value summary from one-way Anova, Tukey's multiple comparisons test

Cytokine	0 vs. 1	0 vs. 2	1 vs. 2
Leptin	0.4389	0.5449	0.9998
GRO	0.7977	0.9997	0.7539
ENA-78	0.8797	0.9969	0.7799
TIMP-2	0.9899	0.9187	0.9283
IGFBP-1	0.1448	0.5826	0.7179
IL-3	0.9005	0.4724	0.5349
IFN-γ	0.8012	> 0.9999	0.8049
FGF-9	0.9545	0.9360	0.9888
IL-16	0.9000	0.9928	0.8121
